# Effects of Hypoxia and Inflammation on Hepcidin Concentration in Non-Anaemic COVID-19 Patients

**DOI:** 10.3390/jcm13113201

**Published:** 2024-05-29

**Authors:** Katarina Gugo, Leida Tandara, Gordana Juricic, Mirela Pavicic Ivelja, Lada Rumora

**Affiliations:** 1 Medical Laboratory Diagnostic Division, University Hospital of Split, Soltanska 1, 21000 Split, Croatia; ltandara@kbsplit.hr; 2 Department of Health Studies, University of Split, Rudera Boskovica 35, 21000 Split, Croatia; mpavivelj@kbsplit.hr; 3 Department of Medical Chemistry and Biochemistry, University of Split School of Medicine, Soltanska 2, 21000 Split, Croatia; 4 Department of Laboratory Diagnostics, General Hospital Pula, Santoriova 24a, 52100 Pula, Croatia; gordana.juricic@obpula.hr; 5 Department of Infectious Diseases, University Hospital of Split, Soltanska 1, 21000 Split, Croatia; 6 Department of Medical Biochemistry and Haematology, University of Zagreb Faculty of Pharmacy and Biochemistry, A. Kovacica 1, 10000 Zagreb, Croatia; lada.rumora@pharma.unizg.hr

**Keywords:** COVID-19, hepcidin, iron, ferritin, erythropoietin, interleukin 6, hypoxia, inflammation

## Abstract

**Background/Objectives:** This study aimed to explore the influence of hypoxia, inflammation, and erythropoiesis on hepcidin and other iron status parameters in non-anaemic COVID-19 patients admitted to the emergency unit before the introduction of therapeutic interventions. **Methods:** Ninety-six COVID-19 patients and 47 healthy subjects were recruited. Patients were subdivided into hypoxic or normoxic groups and, after follow-up, into mild and moderate, severe or critical disease severity groups. Iron, unsaturated iron-binding capacity (UIBC), ferritin, C-reactive protein (CRP), and interleukin 6 (IL-6) were measured on automatic analysers. ELISA kits were used for hepcidin and erythropoietin (EPO) determination. We calculated total iron-binding capacity (TIBC) and ratios of hepcidin with parameters of iron metabolism (ferritin/hepcidin, hepcidin/iron), inflammation (hepcidin/CRP, hepcidin/IL-6), and erythropoietic activity (hepcidin/EPO). **Results:** Hepcidin, ferritin, EPO, CRP, IL-6, ferritin/hepcidin, and hepcidin/iron were increased, while UIBC, TIBC, hepcidin/CRP, and hepcidin/IL-6 were decreased in hypoxic compared to normoxic patients as well as in patients with severe or critical disease compared to those with mild and moderate COVID-19. Regarding predictive parameters of critical COVID-19 occurrence, in multivariable logistic regression analysis, a combination of EPO and ferritin/hepcidin showed very good diagnostic performances and correctly classified 88% of cases, with an AUC of 0.838 (0.749–0.906). **Conclusions:** The hypoxic signal in our group of patients was not strong enough to overcome the stimulating effect of inflammation on hepcidin expression. EPO and ferritin/hepcidin might help to identify on-admission COVID-19 patients at risk of developing a critical form of the disease.

## 1. Introduction

The liver hormone hepcidin is the master regulator of iron metabolism. Hepcidin modulates iron homeostasis through interaction with ferroportin, the only known iron exporter, resulting in internalisation, degradation, and permanent removal of ferroportin from the cell membrane [1]. This leads to the retention of iron in the storage compartments and decreased availability of iron in the extracellular milieu [2,3]. Thus, hepcidin regulates intestinal iron absorption and release of iron from storage compartments. Iron is essential for all living organisms, including microorganisms, which also need iron for survival, replication, and virulence [4]. Consequently, the regulation of iron homeostasis during infection is of crucial importance since both the host and pathogen compete for iron [5,6]. Hepcidin production is regulated on a transcriptional level. Infection, inflammation, and high iron stores stimulate hepcidin expression, and anaemia/hypoxia, low iron stores, and erythropoietic activity downregulate hepcidin expression [7,8]. A rise in hepcidin level leads to iron redistribution to protect the host cells from invading microorganisms through a process called nutritional immunity [9].

Coronavirus disease-2019 (COVID-19) caused by Severe Acute Respiratory Syndrome Coronavirus-2 (SARS-CoV-2) predominantly impacts the respiratory system, though other organs can also be affected. SARS-CoV-2 infection presents with a spectrum of symptoms, ranging from asymptomatic infection to symptomatic disease of different severity [10]. Diverse symptoms can be observed in patients at the time of clinical presentation, with no clear association with risk factors and SARS-CoV-2 genotypes noticed [11]. In severe cases, pulmonary dysfunction occurs, leading to hypoxemia [12]. In patients with SARS-CoV-2 infection, the inflammatory response involves the secretion of various proinflammatory cytokines [13], some of which may impact iron homeostasis. Given that iron availability can influence viral replication [14], host immune effector mechanisms [5], contribute to tissue damage, and worsen disease outcomes [15], potential therapeutic interventions aimed at regulating iron homeostasis in infectious diseases are emerging as promising tools. Therefore, a better understanding of iron homeostasis is necessary.

Studies investigating the parameters of iron homeostasis during the infection with SARS-CoV-2 in COVID-19 found that iron metabolism is altered, and these disturbances mainly manifest as hypoferremia and hyperferritinemia [16,17]. Some authors even propose that iron metabolism changes could be involved in complications of COVID-19-induced pathology [18]. However, it is not entirely clear whether these changes primarily reflect a physiological response to infection or disturbances of iron metabolism actively contribute to COVID-19 pathology [19]. During infection, an elevation in hepcidin level caused primarily by the stimulatory effect of interleukin 6 (IL-6) leads to the development of hypoferremia [20]. However, in some pathological conditions like COVID-19, different factors which control hepcidin expression can be simultaneously present, generating opposing signals for hepcidin expression. It was shown that the final effect of signals that regulate hepcidin expression is not determined only by the hierarchy between signalling pathways but rather by the strength of the individual stimuli [21]. Studies that measured hepcidin concentration in the circulation of COVID-19 patients gave conflicting results, with most studies demonstrating elevated hepcidin concentrations [22,23,24,25], but its decreased concentrations in critically ill patients compared to healthy controls were also reported [26].

While most studies have primarily focused on the prognostic value of iron status biomarkers [16,22,23,27], insufficient studies regarding the mechanisms regulating iron metabolism in SARS-CoV-2 infection are available. Hypoxia and inflammation are simultaneously present in COVID-19 as two opposing signals which influence hepcidin expression. Studies on human models involving healthy volunteers have shown that hypoxia can prevail over inflammatory stimuli on hepcidin expression [28,29]. Hypoxia might regulate hepcidin expression through the induction of hypoxia-inducible transcription factors. Hypoxia also stimulates erythropoietin (EPO) production, which stimulates erythropoiesis and leads to the expression of erythroferrone in erythroblasts [30,31], and erythroferrone probably directly inhibits hepcidin expression in response to increased erythropoietic demand for iron [32].

Our hypothesis is that in COVID-19 patients, hypoxia, when present, might overcome the inflammatory impact on hepcidin expression and cause a decrease in its concentration in peripheral blood compared to COVID-19 patients with normal oxygen saturation. The aim of this study was to explore the influence of hypoxia, inflammation, and erythropoiesis on concentrations of hepcidin and other parameters of iron status in groups of non-anaemic normoxic and hypoxic COVID-19 patients on admission into the emergency unit before the introduction of therapeutic interventions. We applied rigorous exclusion criteria to eliminate comorbidities and conditions that could influence hepcidin concentration. We also examined the diagnostic potential of various iron metabolism-related parameters measured on admission to predict the risk of developing a critical form of the disease later on.

Therefore, we have determined the concentrations of hepcidin as well as the parameters of iron metabolism, inflammation, and erythropoiesis. We also calculated various ratios of hepcidin with parameters of iron metabolism (ferritin-to-hepcidin ratio; hepcidin-to-iron ratio) and parameters reflecting inflammation (hepcidin-to-CRP ratio; hepcidin-to-IL-6 ratio) or erythropoietic activity (hepcidin-to-EPO ratio).

## 2. Materials and Methods

### 2.1. Study Subjects

This study included 47 healthy volunteers as a control group, and 96 COVID-19 patients with COVID-19 symptoms were admitted to the emergency unit of the Infectious Disease Department of the University Hospital of Split (Split, Croatia). SARS-CoV-2 positivity was confirmed by reverse transcription polymerase chain reaction (RT-PCR) from a nasopharyngeal swab. The inclusion criterion was age between 20 and 75 years for both groups. Exclusion criteria applied were as follows: anaemia (haemoglobin < 120 g/L for women and <130 g/L for men); history of any haematological disease; liver disease; chronic kidney disease; estimated glomerular filtration rate (eGFR) < 60 mL/min; inflammatory bowel disease; systemic autoimmune disease; malignant disease; chronic obstructive pulmonary disease; asthma; surgical procedure and blood transfusion in previous 3 months; therapy with iron supplements; pregnancy; and breastfeeding.

Based on oxygen saturation levels measured on admission, COVID-19 patients were classified into two groups: hypoxic (SpO_2_ < 94%); and normoxic (SpO_2_ ≥ 94%). A total of 47 hypoxic COVID-19 patients and 49 normoxic COVID-19 patients were matched by age and sex. The control group was also matched by age and sex to the COVID-19 patients and met the same criteria, except for SARS-CoV-2 positivity.

COVID-19 patients were categorised based on prospective follow-up into three groups, according to the World Health Organization guidelines [33]: patients with mild and moderate, severe and critical COVID-19. Symptomatic COVID-19 patients without viral pneumonia or hypoxia were classified as mild cases. Moderate COVID-19 disease was determined by the presence of mild pneumonia with SpO_2_ > 90% on room air, while severe disease was indicated by severe pneumonia with SpO_2_ < 90% on room air. Critical COVID-19 disease was identified by respiratory failure resulting from viral pneumonia and other critical conditions requiring mechanical respiratory support and other life-sustaining therapies.

### 2.2. Study Design

This single-centre observational study was performed at the University Hospital of Split between June 2021 and June 2022. This study was approved by the Ethics Committee of the University Hospital of Split (Split, Croatia) and Ethics Committee for Experimentation of the University of Zagreb Faculty of Pharmacy and Biochemistry (Zagreb, Croatia) (approval protocol numbers: 2181-147/01/06/M.S.-21-02 and 251-62-03-23-58, respectively) and was conducted according to the principles of the Declaration of Helsinki. Written informed consent was obtained from all study participants.

For each patient demographic, clinical and anamnestic data were collected upon arrival at the emergency unit and analysis of oxygen saturation (SpO_2_), complete blood count, and routine biochemical tests were performed. Information on the course of the disease was prospectively monitored by reviewing the medical documentation from the hospital information system.

In addition to routine tests performed on admission, the concentrations of hepcidin, iron, unsaturated iron-binding capacity (UIBC), ferritin, soluble transferrin receptors (sTfR), reticulocyte haemoglobin equivalent (RET-He), reticulocyte number (RTC), immature reticulocyte fraction (IRF), EPO, C-reactive protein (CRP), and IL-6 were also measured for all subjects, and total iron-binding capacity (TIBC) and transferrin saturation (TSAT) were calculated, as well as various ratios that included hepcidin (ferritin/hepcidin, hepcidin/iron, hepcidin/CRP, hepcidin/IL-6, and hepcidin/EPO).

### 2.3. Blood Sampling

Blood samples were taken immediately upon admission of the COVID-19 patients to the emergency unit of the Department of Infectious Disease prior to the initiation of therapeutic interventions. Blood sampling for the control group was performed at the Medical Laboratory Diagnostic Division of the University Hospital of Split (Split, Croatia).

Two tubes of venous blood were sampled for all patients: a tube with K3-ethylenediaminetetraacetic acid (K3EDTA); and a tube with lithium heparin (Beckton Dickinson, Frankin Lakes, NJ, USA). Arterial blood samples for determination of SpO_2_ were taken from the radial artery.

Plasma sample for biochemical analyses was obtained by centrifugation of lithium heparin anticoagulated sample at 1800× *g* for 10 min. All routine analyses were performed within 1 h of receiving the samples at the laboratory, and the remaining plasma samples were immediately aliquoted and stored at –80 °C until analyses.

### 2.4. Methods

Haemoglobin concentration, RTC number, IRF, and RET-He were measured on the haematology analyser Sysmex XN-1000 (Sysmex Corporation, Kobe, Japan). Plasma concentration of CRP, creatinine, iron, UIBC, ferritin, and IL-6 was measured by standard laboratory methods on a Roche Cobas 6000 analyser (Roche Diagnostics GmbH, Mannheim, Germany). Since IL-6 concentration was below the reported range in some individuals, a nominal level of half of the lower limit of quantification (LLQ) value (1.25 pg/mL) was used in the analysis in these individuals [34]. TIBC and TSAT were calculated by the following equations: TIBC = iron (µmol/L) + UIBC (µmol/L); TSAT (%) = iron (µmol/L)/TIBC (µmol/L) × 100. eGFR was calculated according to the CKD-EPI (Chronic Kidney Disease Epidemiology Collaboration) equation. sTfR concentration was measured nephelometrically on a BN ProSpec analyser (Siemens Healthcare Diagnostics, Marburg, Germany). All parameters were determined at the University Hospital of Split (Split, Croatia) except hepcidin and EPO, which were measured at the University of Zagreb Faculty of Pharmacy and Biochemistry (Zagreb, Croatia). Hepcidin and EPO concentrations were determined by commercially available ELISA tests: Hepcidin 25 (bioactive) HS ELISA (DRG Diagnostics GmbH, Marburg, Germany); and Quantikine^®^ IVD^®^ Human Erythropoietin ELISA (R&D Systems Inc., Minneapolis, MN, USA) according to manufacturers’ instructions. SpO_2_ was measured by an oximetric method on an ABL90 analyser FLEX PLUS (Radiometer, Copenhagen, Denmark).

### 2.5. Statistical Analysis

All data were tested for normality of distribution with Shapiro–Wilk test. Depending on the data distribution, results were presented as mean ± standard deviation for data showing normal distribution, otherwise as median with Q1–Q3 range. Age was shown as median with minimum and maximum. Categorical variables were presented as an absolute number N. A comparison of categorical variables between two groups was performed by Fisher’s exact test and between three groups by Chi-squared test. The difference between the two groups was tested using a *t*-test or Mann–Whitney test. Differences between more than two groups were tested using a one-way ANOVA analysis of variance or the Kruskal–Wallis test with post hoc testing. Univariate and multivariate logistic regression analyses were performed. Data were considered significant if *p* < 0.05. Data analysis was performed using the MedCalc statistical software version 20.013 (MedCalc Software, Ostend, Belgium).

## 3. Results

The research included 96 COVID-19 patients and 47 age- and sex-matched healthy subjects. Patients with COVID-19 disease were classified as normoxic or hypoxic on admission to the emergency unit based on their SpO_2_ values. Baseline characteristics of healthy volunteers and COVID-19 patients on admission are presented in Table 1.

Hypertension was the most common comorbidity in all study groups, occurring in 15/47 hypoxic COVID-19 patients, 7/49 normoxic COVID-19 patients, and 14/47 healthy individuals. The incidence of diabetes mellitus was low in our study participants, with 2/47 in the hypoxic group, 4/49 in the normoxic group, and 6/47 in the control group. Out of a total of 96 COVID-19 patients, 53 were hospitalised upon admission to the emergency unit. Those included all hypoxic patients with a median hospitalisation duration of 9 (7–18) days and six normoxic patients with a median hospitalisation duration of 4 (3–5) days. Nineteen hypoxic patients, all with critical disease severity, subsequently required mechanical ventilation, and only six of them died. Additionally, there was no statistically significant difference in the concentration of creatinine and eGFR among the studied groups (*p* = 0.46 and *p* = 0.70, respectively).

The clinical manifestation data of patients with COVID-19 on admission are presented in Table 2.

In order to investigate a potential disturbance in hepcidin expression in COVID-19 patients and the influence of factors that might affect it, we measured hepcidin concentration in peripheral blood as well as concentrations of iron metabolism parameters, parameters reflecting erythropoietic activity, and parameters of systemic inflammation.

**Figure 1:**
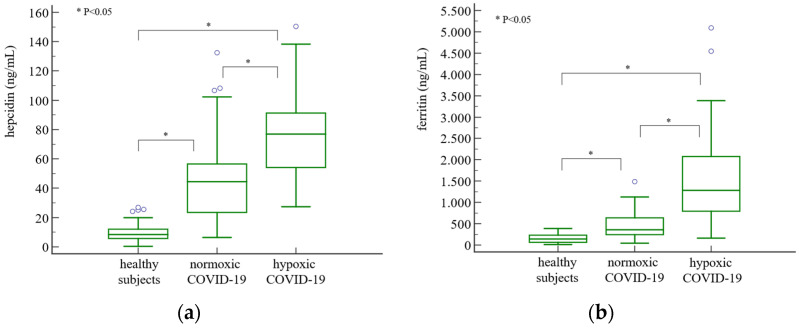

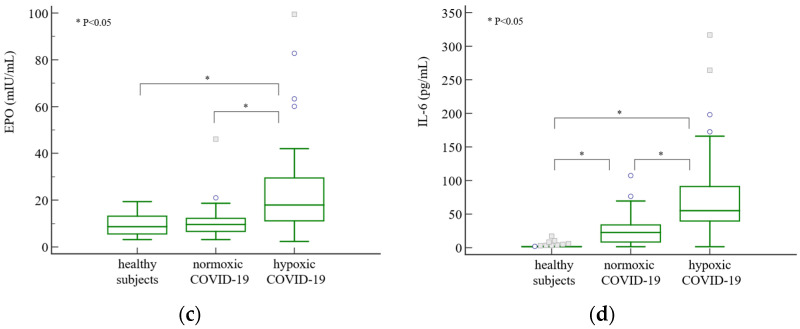
Hepcidin (**a**), ferritin (**b**), EPO (**c**), and IL-6 (**d**) plasma concentrations in healthy subjects and in normoxic and hypoxic COVID-19 patients. Results are shown as the median with Q1–Q3 range. The difference between groups was tested using the Kruskal–Wallis test with post hoc analysis (Conover). Connectors on the graphs show between which groups a statistically significant difference was observed in post hoc analysis with *p* < 0.05. EPO, erythropoietin; IL-6, interleukin 6.

Hepcidin concentrations were significantly higher in both normoxic [44.50 (23.49–56.54) ng/mL] and hypoxic [76.95 (54.14–91.28) ng/mL] groups of COVID-19 patients compared to healthy controls [8.38 (5.74–12.06) ng/mL] (*p* < 0.001), with the highest values in the hypoxic group (Figure 1a). Similarly, ferritin was also significantly increased in COVID-19 patients, especially in those with hypoxia (Figure 1b).

Iron, TIBC, TSAT, RET-He, RTC, and IRF were decreased, and sTfR was higher in both normoxic and hypoxic patients compared to healthy individuals, while UIBC and TIBC were lower, and IRF was higher in the hypoxic group compared to the normoxic group (Table 3). EPO was increased only in the hypoxic group compared to controls and normoxic patients (Figure 1c).

Both CRP and IL-6 were higher in patients with COVID-19 disease, especially in those with hypoxia (Table 3 and Figure 1d). Finally, values for ferritin/hepcidin and hepcidin/iron ratios were elevated, while values for hepcidin/CRP and hepcidin/IL-6 ratios were lower in the hypoxic compared to the normoxic group (Table 3).

Later on, patients infected with SARS-CoV-2 were also classified by disease severity based on following up their medical history. Only two patients with hypoxia were classified in the mild and moderate group (*n* = 49), while 26 of 28 patients in the severe group were hypoxic, and all 19 patients in the critical group were hypoxic on admission.

**Figure 2:**
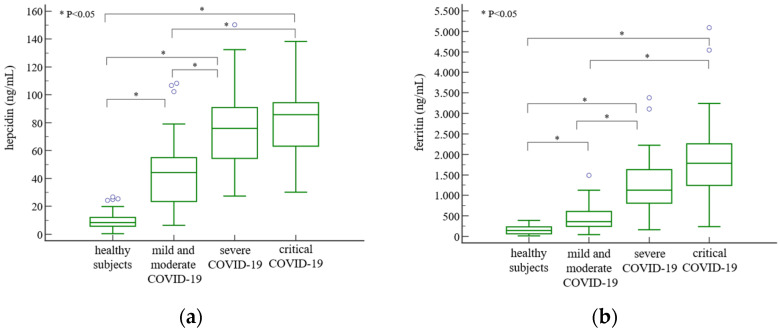

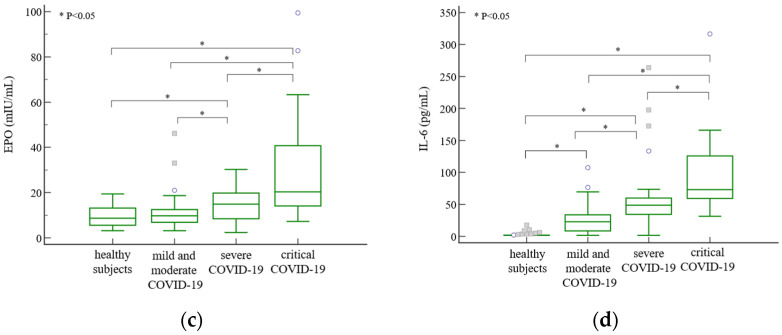
Hepcidin (**a**), ferritin (**b**), EPO (**c**), and IL-6 (**d**) plasma concentrations in healthy subjects and in COVID-19 patients subdivided by disease severity by prospective follow-up. Results are shown as the median with Q1–Q3 range. The difference between groups was tested using the Kruskal–Wallis test with post hoc analysis (Conover). Connectors on the graphs show between which groups a statistically significant difference was observed in post hoc analysis with *p* < 0.05. EPO, erythropoietin; IL-6, interleukin 6.

Hepcidin concentrations were significantly increased in patients with mild and moderate [44.25 (23.49–54.97) ng/mL], severe [75.95 (54.40–90.91) ng/mL], and critical [85.79 (63.18–94.38) ng/mL] disease course compared to healthy subjects (*p* < 0.001) and were higher in severe and critical patients compared with patients in mild and moderate COVID-19 groups (Figure 2a). Concentrations of ferritin were also the highest in severe and critical patients (Figure 2b), as well as concentrations of EPO (Figure 2c), IL-6 (Figure 1d), and CRP (Table 4) compared to patients with mild or moderate disease severities.

No significant differences between disease severity groups were observed for iron concentration, TSAT, sTfR, RET-He, and RTC. UIBC, TIBC, hepcidin/CRP, and hepcidin/IL-6 were lower, and IRF, ferritin/hepcidin, and hepcidin/iron were higher in severe and critical COVID-19 than in mild and moderate COVID-19 (Table 4). Only EPO, IL-6, and hepcidin/EPO differentiated patients with critical COVID-19 from those with severe disease course.

Next, predictive values of investigated parameters for critical COVID-19 disease were assessed by univariate logistic regression analysis (Table 5). Hepcidin, ferritin, EPO, CRP, IL-6, ferritin/hepcidin, and hepcidin/iron appear to be predictors of the risk for critical disease severity, while UIBC and TIBC have reduced odds.

Finally, we performed a multivariate logistic regression analysis, and we tested the multiparameter model that included the best predictors of the risk for critical COVID-19 (Table 6).

## 4. Discussion

This study assessed the influence of hypoxia, inflammation, and erythropoietic activity on hepcidin and other iron homeostasis-related parameters in COVID-19 patients. In addition, in an attempt to identify representative surrogates that could detect dysregulation of iron homeostasis as well as differentiate between various disease subgroups, we included combinations of parameters (ratios) that might take advantage of the relationships between different phenomena (iron status, inflammation, erythropoiesis).

The ratio between hepcidin and ferritin was used in only two studies that compared COVID-19 patients with patients with other infectious and non-infectious conditions, and those studies recruited only 40 COVID-19 patients in total [35,36]. In addition, ferritin-to-hepcidin or hepcidin-to-ferritin ratios were used to investigate other diseases as well [37,38,39]. Regarding hepcidin/iron and hepcidin/EPO ratios, they have not been explored in COVID-19 so far but in some other diseases [39,40,41,42]. Hepcidin/CRP was applied in only one study with subjects infected with SARS-CoV-2 [36]. We also introduced for the first time hepcidin/IL-6, as IL-6 is the main cytokine inducer of hepcidin expression, and we wanted to test this ratio and compare it to hepcidin/CRP in the context of normalisation of hepcidin concentration with existing inflammation.

Our results show that hepcidin, as well as ferritin, IRF, EPO, CRP, IL-6, ferritin/hepcidin, and hepcidin/iron, were significantly increased, while UIBC, TIBC, hepcidin/CRP, and hepcidin/IL-6 were significantly decreased in hypoxic compared to normoxic patients as well as in patients with severe or critical disease compared to those with mild and moderate COVID-19. In addition, EPO, IL-6, and hepcidin/EPO ratio were able to differentiate subjects with critical and severe disease courses.

Our results are in line with previously reported studies, which found elevated hepcidin concentration in COVID-19 patients. In the study of Zhou et al. [23], hepcidin and ferritin concentrations were higher in severe than mild group, and authors suggested that they could be, therefore, used as markers of disease severity. However, no information was available on the degree of hypoxemia in these patient groups, and they did not measure CRP or IL-6 inflammatory markers. In a prospective study by Chakurkar et al. [15], concentrations of hepcidin, ferritin, and CRP, yet not IL-6, were dependent on disease severity and were the highest in severe compared to mild and moderate COVID-19 groups. In addition, baseline levels of hepcidin and ferritin were found to be associated with negative outcomes of disease, such as in-hospital mortality and the need for mechanical ventilation and kidney replacement therapy. In the study of Nai et al. [22], hepcidin showed a positive correlation with CRP and a negative correlation with the severity of respiratory failure as reflected by the PaO_2_/FiO_2_ ratio, and hepcidin was proposed as a predictor of mortality of critical patients in the intensive care unit. Similarly to our results, they also found that hepcidin and CRP concentrations were higher in the hypoxemic group (PaO_2_/FiO_2_ ≤ 300 mm Hg) compared to the normoxemic group (PaO_2_/FiO_2_ > 300 mm Hg); however, IL-6 level did not differ between those two groups.

Lower hepcidin concentration was reported in critically ill COVID-19 patients than in healthy controls [26]. In the same research, lower concentrations of EPO in critical and deceased patients were found, and the critical patient group had significantly lower haemoglobin values than other study groups. No information about inflammatory markers was provided. The authors proposed that low hepcidin concentration could be explained by the hepcidin mimetic action of the SARS-CoV-2 virus that could cause suppression of hepcidin expression in the liver. This theory is based on sequence similarity between the hepcidin molecule and the SARS-CoV-2 spike glycoprotein cytoplasmic tail, which was found in structural models [43]. Contrary to our results, in a study by Maira et al. [44] involving hospitalised COVID-19 patients, when patients were categorised by severity of hypoxemia on admission (PaO_2_/FiO_2_ < 150 mmHg vs. PaO_2_/FiO_2_ ≥ 150 mmHg), no difference in hepcidin and EPO was observed between groups at admission to the ward or after 7 days of hospitalisation, although the hypoxemic group had a higher level of inflammatory markers. The authors stated that the possible reason for the inadequate rise in EPO could be due to the inflammatory inhibition of EPO production by kidneys. In conclusion, the authors suggested that hypoxic stimuli suppressed the effect of the inflammatory signal on hepcidin expression and led to lower hepcidin values in patients with a higher disease burden. It is also worth mentioning that a large proportion of study participants was anaemic.

The design of studies might have a significant effect on the results obtained. A possible reason for lower hepcidin levels could also be the presence of anaemia. In our study, all anaemic patients were excluded to eliminate the possible effect of pre-existing iron deficiency and anaemia on hepcidin expression since our aim was to study only the simultaneous effects of inflammation and hypoxemia caused by respiratory failure. Therefore, in the hypoxic group of patients, the effect of inflammation on hepcidin level might be somewhat attenuated by the simultaneous presence of hypoxia as an opposing signal.

In that light, we aimed to express the hepcidin level relative to the level of inflammation by calculating the ratio of hepcidin with IL-6 as the main proinflammatory cytokine involved in the direct stimulation of hepcidin expression. We suggest this ratio may be applied in diseases and/or conditions where inflammation is present and not in those without inflammation accompanied by synthesis of IL-6 (e.g., in healthy subjects). In our study, the hepcidin/IL-6 ratio was lower in the hypoxic than in the normoxic group, as well as in severe and critical COVID-19 (where all patients except two were hypoxic), which could further indicate that hepcidin level was not proportional to the level of inflammation in the hypoxic patient group probably due to downregulating effect of hypoxia. When IL-6 was replaced with CRP as a routinely measured inflammatory marker, the same result was obtained with the hepcidin/CRP ratio.

In our hypoxic group of patients, a significantly higher concentration of EPO was found, which reflects the normal response to hypoxia. Nevertheless, no simultaneous reduction in hepcidin level was observed. Studies on animal models have shown that erythropoiesis itself, rather than EPO alone, is needed to suppress hepcidin expression [45]. In our study groups, erythropoietic activity was equally suppressed in both hypoxic and normoxic patients compared to healthy subjects, as shown by lower reticulocyte numbers. More pronounced inflammation observed in the hypoxic group might have led to an inadequate EPO response, consequently resulting in the absence of an increase in reticulocyte count. Since we did not measure erythroferrone concentration, we cannot speculate whether the rise in EPO concentration was followed by an adequate rise in the concentration of this hormone. During maturation, reticulocytes gradually lose their RNA and become RNA-free cells, while a small proportion of reticulocytes containing higher levels of RNA represent IRF. IRF is proposed to be a marker of erythropoiesis acceleration since immature reticulocytes become more prevalent when red cell production increases, and an increase in IRF typically occurs several days before the rise in RTC count as a quantitative measure of erythropoiesis [46]. In our study, we observed decreased IRF values in COVID-19 patient groups compared to healthy controls. Interestingly, despite this decrease, values were higher in hypoxic compared to normoxic COVID-19 patients. This observation suggests a potential counteracting effect of EPO on erythropoietic activity already compromised by the presence of severe inflammation.

Moreover, given the nature of our study, information regarding the duration of hypoxia in our patients is unknown, which could be one of the reasons why suppression in hepcidin level was not observed. The time delay in hepcidin suppression following exposure to hypoxia highlights the significance of indirect mechanisms in mediating hepcidin expression under hypoxic conditions. In a study conducted on healthy volunteers exposed to acute and chronic high-altitude conditions, a rapid increase in EPO concentration was noted. However, suppression of hepcidin began a few hours after initial exposure to acute hypoxia and reached its lowest points during the following week of high-altitude exposure [28]. Likewise, in a study on healthy volunteers, hepcidin suppression was observed 24 h after EPO administration [47]. Studies on animal models with experimentally induced anaemia have indicated that suppression of hepcidin expression is dependent on erythropoiesis and not directly mediated by hypoxia, anaemia, or EPO [48].

In our study, high ferritin concentration was found in all COVID-19 patients, with significantly higher values in the hypoxic compared to the normoxic group. High ferritin concentration was also found in severe and critical COVID-19 compared to mild and moderate COVID-19 groups. Higher ferritin concentration in more severe COVID-19 disease was confirmed in a number of studies [23,26,49]. High ferritin concentration is considered to be not only a marker of disease but also a prognostic factor in COVID-19 disease [15]. In a study by Sonnweber and colleagues [17], ferritin concentration was elevated even 2 months after critical COVID-19 and was associated with persisting lung pathologies and decreased physical performance. Ferritin concentration increases as a result of cellular iron accumulation influenced by the effects of hepcidin [30]. In addition, both hepcidin and ferritin are not only regulated by iron status but are significantly influenced by inflammatory stimuli [2,5]. In our study, ferritin concentration positively correlated with hepcidin concentration, as already observed in COVID-19 patients [35]. Therefore, we applied the ferritin/hepcidin ratio to simultaneously follow their dynamics in different COVID-19 contexts (regarding oxygen saturation and disease severity). This ratio was increased in hypoxic compared to normoxic patients as well as in patients with severe and critical compared to mild and moderate diseases.

In this research, in order to evaluate the predictive potential of examined parameters, we performed univariate logistic regression analysis. This analysis indicated increased odds for critical COVID-19 severity for hepcidin, ferritin, EPO, CRP, IL-6, ferritin/hepcidin, and hepcidin/iron, while UIBC and TIBC had the OR of less than 1.

Next, we performed a multivariable logistic regression analysis that included only parameters that showed to be the best predictors for the risk of developing critical disease. The combination of EPO and ferritin/hepcidin ratio showed very good diagnostic performances and correctly classified 88% of cases, with an AUC of 0.838 (0.749–0.906). Implementation of EPO and ferritin/hepcidin in clinical practice might help to identify on-admission COVID-19 patients who are at risk of developing critical forms of disease.

The limitation of our study was the unstandardised time of blood withdrawal, which was inevitable because samples were taken immediately upon admission to the emergency unit, and this could influence concentrations of some parameters with diurnal variation. However, we also consider this to be one of the strengths of our study because samples taken on admission provide us with real concentrations of the investigated parameters before the introduction of oxygen therapy and other interventions or in-hospital complications, which could confound our results. Despite the majority of samples being collected during the prevalence of the Delta genotype of the SARS-CoV-2 virus, we believe that this should not impact the study results. Our focus was not on immune mechanisms but rather on using COVID-19 as a model of disease where hypoxia and inflammation coexist as opposing factors influencing hepcidin concentration.

## 5. Conclusions

To the best of our knowledge, this is the first study that investigated the simultaneous presence of inflammation and hypoxia in non-anaemic COVID-19 patients selected based on rigorous exclusion criteria in order to eliminate comorbidities and conditions that might influence concentrations of the studied parameters. In addition, hepcidin/iron and hepcidin/EPO ratios have not been previously explored in patients with SARS-CoV-2 infection, and we also introduced the hepcidin/IL-6 ratio to observe, at the same time, hepcidin concentration normalised for the level of inflammation. We can conclude that the hypoxic signal in our group of patients was not strong enough to overcome the stimulating effect of inflammation on hepcidin expression. These results imply that hepcidin targeting could be a promising therapeutic option worth exploring in order to affect the iron metabolism in these patients.

## Figures and Tables

**Table 1:** Baseline patient characteristics.

	Healthy Subjects (*n* = 47)	Total COVID-19 Patients (*n* = 96)	Normoxic COVID-19 Patients (*n* = 49)	Hypoxic COVID-19 Patients (*n* = 47)	P_1_	P_2_	P_3_
age (years)	58 (39–70)	54 (36–73)	53 (36–73)	55 (37–72)	0.19	0.17	0.18
sex male, *n* female, *n*	31 16	64 32	31 18	33 14	1.00	0.77	0.52
SpO_2_ (%)	/	96 (88–98)	97 (97–98)	87 (83–89)	/	/	<0.001
creatinine (µmol/L)	77 ± 12	79 ± 14	80 ± 15	78 ± 14	0.29	0.46	0.48
eGFR (mL/min/1.73 m^2^)	91 (80–99)	87 (77–101)	86 (78–99)	88 (77–102)	0.52	0.70	0.61
symptoms duration on admission (days), *n*	/	8 ± 3	8 ± 3	9 ± 3	/	/	0.001

SpO_2_, oxygen saturation. eGFR, estimated glomerular filtration rate. P_1_—statistical significance of differences between healthy subjects and total COVID-19 patients. P_2_—statistical significance of differences between healthy subjects, normoxic COVID-19 patients and hypoxic COVID-19 patients; post hoc analysis was performed. P_3_—statistical significance of differences between normoxic and hypoxic COVID-19 patients. Age was presented as median with minimum and maximum, while sex was presented as absolute number *n*. SpO_2_ and eGFR were presented as median with Q1–Q3 range and creatinine concentration and symptoms duration on admission as mean ± standard deviation. Data were considered significant if *p* < 0.05.

**Table 2:** The clinical manifestations of patients with COVID-19 on admission.

Clinical Manifestations	Normoxic COVID-19 Patients (*n* = 49)	Hypoxic COVID-19 Patients (*n* = 47)
fever before admission	47 (96)	46 (98)
fever on admission	28 (57)	35 (74)
cough	35 (71)	36 (77)
sore throat	9 (18)	8 (17)
dyspnea	0 (0)	35 (74)
fatigue	19 (39)	32 (68)
myalgia	18 (37)	17 (36)
pneumonia on X-ray imaging	16 (33)	47 (100)

The results are presented as absolute number *n* (percentage, %).

**Table 3:** Parameters of iron homeostasis, erythropoietic activity, and inflammation in total, normoxic and hypoxic COVID-19 patients, as well as in healthy subjects.

Parameter	Healthy Subjects (*n* = 47)	Total COVID-19 Patients (*n* = 96)	Normoxic COVID-19 Patients (*n* = 49)	Hypoxic COVID-19 Patients (*n* = 47)	P_1_	P_2_
haemoglobin (g/dL)	15.0 (13.7–15.4)	14.5 (13.5–15.4)	14.4 (13.5–15.6)	14.6 (13.6–15.4)	0.48	0.75
iron (µmol/L)	17.0 (13.3–21.0)	6.0 (5.0–8.5)	6.0 (5.0–8.3) ^a^	5.0 (4.3–8.8) ^a^	<0.001	<0.001
UIBC (µmol/L)	42 (35–49)	39 (32–45)	44 (37–47)	33 (29–40) ^a,b^	0.06	<0.001
TIBC (µmol/L)	59 (54–68)	46 (40–51)	50 (45–54) ^a^	42 (37–46) ^a,b^	<0.001	<0.001
TSAT (%)	28 (23–36)	13 (10–18)	12 (10–16) ^a^	15 (10–20) ^a^	<0.001	<0.001
sTfR (mg/L)	1.09 (1.00–1.25)	1.26 (1.07–1.45)	1.30 (1.12–1.49) ^a^	1.23 (1.02–1.41) ^a^	0.002	0.002
RET-He (pg)	33.0 (32.4–33.7)	30.1 (28.6–31.5)	30.2 (28.8–31.0) ^a^	30.1 (28.4–31.9) ^a^	<0.001	<0.001
RTC (‰)	13.20 (11.70–15.58)	5.25 (4.40–6.85)	5.00 (4.18–6.98) ^a^	5.30 (4.43–6.80) ^a^	<0.001	<0.001
RTC (×10^9^/L)	67.60 (56.05–82.05)	26.10 (20.90–34.80)	25.60 (19.45–35.05) ^a^	26.20 (21.80–34.63) ^a^	<0.001	<0.001
IRF (%)	8.9 (7.5–10.3)	4.6 (3.4–7.9)	3.6 (3.0–4.9) ^a^	6.2 (4.1–10.7) ^a,b^	<0.001	<0.001
CRP (mg/L)	1.2 (0.7–2.3)	37.1 (12.7–100.0)	13.1 (5.2–33.2) ^a^	85.5 (39.7–120.7) ^a,b^	<0.001	<0.001
ferritin/hepcidin	15.17 (9.84–25.09)	13.55 (7.06–20.91)	7.72 (6.24–13.98) ^a^	19.02 (12.98–29.64) ^b^	0.10	<0.001
hepcidin/iron	0.49 (0.34–0.84)	10.24 (5.04–14.05)	7.37 (3.44–12.00) ^a^	13.04 (8.06–16.04) ^a,b^	<0.001	<0.001
hepcidin /CRP	7.14 (2.70–12.16)	1.67 (0.77–3.25)	2.58 (1.39–6.42) ^a^	0.93 (0.53–1.80) ^a,b^	<0.001	<0.001
hepcidin/IL-6	4.54 (2.50–7.02)	1.57 (1.00–2.45	1.94 (1.42–3.69) ^a^	1.33 (0.61–1.92) ^a,b^	<0.001	<0.001
hepcidin/EPO	1.13 (0.57–2.14)	4.09 (2.32–7.60)	4.23 (2.36–7.81) ^a^	4.08 (2.26–7.37) ^a^	<0.001	<0.001

UIBC, unsaturated iron-binding capacity; TIBC, total iron-binding capacity; TSAT, transferrin saturation; sTfR, soluble transferrin receptors; RET-He, reticulocyte haemoglobin equivalent; RTC, reticulocytes; IRF, immature reticulocyte fraction; CRP, C-reactive protein; IL-6, interleukin 6; EPO, erythropoietin. P_1_—statistical significance of differences between healthy subjects and total COVID-19 patients. P_2_—statistical significance of differences between healthy subjects, normoxic COVID-19 patients and hypoxic COVID-19 patients; afterwards, post hoc analysis was performed. ^a^ statistically significant difference between normoxic or hypoxic COVID-19 patients and healthy subjects. ^b^ statistically significant difference between normoxic and hypoxic COVID-19 patients. Results are presented as median with Q1–Q3 range. Data were considered significant if *p* < 0.05.

**Table 4:** Parameters of iron homeostasis, erythropoietic activity and inflammation in healthy subjects and COVID-19 patients subdivided prospectively by disease severity into groups of mild and moderate, severe, and critical COVID-19 disease.

Parameter	Healthy Subjects (*n* = 47)	Mild and Moderate COVID-19 (*n* = 49)	Severe COVID-19 (*n* = 28)	Critical COVID-19 (*n* = 19)	*p*
haemoglobin (g/dL)	15.0 (13.7–15.4)	14.3 (13.4–15.6)	14.5 (13.8–15.4)	14.7 (13.6–15.2)	0.87
iron (µmol/L)	17.0 (13.3–21.0)	6.0 (5.0–8.0) ^a^	6.0 (5.0–9.0) ^a^	5.0 (4.3–8.8) ^a^	<0.001
UIBC (µmol/L)	42 (35–49)	44 (36–47)	34 (29–39) ^a,b^	33 (29–41) ^a,b^	<0.001
TIBC (µmol/L)	59 (54–68)	49 (44–53) ^a^	43 (38–47) ^a,b^	41 (37–47) ^a,b^	<0.001
TSAT (%)	28 (23–36)	12 (10–15) ^a^	15 (11–19) ^a^	16 (10–21) ^a^	<0.001
sTfR (mg/L)	1.09 (1.00–1.25)	1.29 (11.12–1.48) ^a^	1.23 (1.06–1.45) ^a^	1.23 (1.01–1.40)	0.010
RET-He (pg)	33.0 (32.4–33.7)	30.2 (28.6–31.0) ^a^	30.1 (28.5–31.8) ^a^	30.1 (28.7–31.6) ^a^	<0.001
RTC (‰)	13.20 (11.70–15.58)	5.20 (4.18–6.98) ^a^	5.45 (4.50–6.80) ^a^	4.90 (4.40–6.63) ^a^	<0.001
RTC (×10^9^/L)	67.60 (56.05–82.05)	25.60 (19.45–35.58) ^a^	26.75 (22.05–35.05) ^a^	26.10 (20.90–33.38) ^a^	<0.001
IRF (%)	8.9 (7.5–10.3)	3.6 (3.1–5.4) ^a^	6.5 (4.4.–11.0) ^a,b^	4.9 (3.5–10.0) ^a,b^	<0.001
CRP (mg/L)	1.2 (0.7–2.3)	14.3 (5.2–35.2) ^a^	84.0 (38.1–120.7) ^a,b^	86.7 (42.1–129.9) ^a,b^	<0.001
ferritin/hepcidin	15.17 (9.84–25.09)	7.72 (6.26–14.19) ^a^	16.78 (11.94–24.23) ^b^	23.16 (13.83–33.23) ^b^	<0.001
hepcidin/iron	0.49 (0.34–0.84)	7.37 (3.44–12.00) ^a^	12.40 (8.08–15.50) ^a,b^	13.04 (9.86–16.92) ^a,b^	<0.001
hepcidin /CRP	7.14 (2.70–12.16)	2.49 (1.34–6.42) ^a^	1.03 (0.61–1.76) ^a,b^	0.87 (0.43–1.98) ^a,b^	<0.001
hepcidin/IL-6	4.54 (2.50–7.02)	1.85 (1.33–3.27) ^a^	1.47 (0.75–2.07) ^a,b^	1.39 (0.59–1.64) ^a,b^	<0.001
hepcidin/EPO	1.13 (0.57–2.14)	4.07 (2.13–6.89) ^a^	5.40 (3.21–7.86) ^a^	3.29 (1.51–6.42) ^a,c^	<0.001

UIBC, unsaturated iron-binding capacity; TIBC, total iron-binding capacity; TSAT, transferrin saturation; sTfR, soluble transferrin receptors; RET-He, reticulocyte haemoglobin equivalent; RTC, reticulocytes; IRF, immature reticulocyte fraction; CRP, C-reactive protein; IL-6, interleukin 6; EPO, erythropoietin. *p*—statistical significance of differences between healthy subjects and COVID-19 patients with different disease severity; afterwards, post hoc analysis was performed. ^a^ statistically significant difference between mild and moderate, severe, or critical COVID-19 patients and healthy subjects. ^b^ statistically significant difference between severe or critical and mild and moderate COVID-19 patients. ^c^ statistically significant difference between severe and critical COVID-19 patients. Results are presented as median with Q1–Q3 range. Data were considered significant if *p* < 0.05.

**Table 5:** Univariate logistic regression analysis of parameters of iron homeostasis, erythropoietic activity, and inflammation.

Odds for Critical COVID-19			
Parameter	OR	95% CI	*p*
haemoglobin (g/L)	1.01	0.97–1.05	0.73
hepcidin (ng/mL)	1.03	1.01–1.05	0.004 *
iron (µmol/L)	0.96	0.83–1.11	0.58
UIBC (µmol/L)	0.92	0.86–0.99	0.028 *
TIBC (µmol/L)	0.91	0.85–0.98	0.013 *
TSAT (%)	1.01	0.95–1.08	0.69
ferritin (ng/mL)	1.00	1.00–1.00	<0.001 *
sTfR (mg/L)	0.93	0.16–5.50	0.94
RET-He (pg)	1.03	0.80–1.33	0.82
RTC (‰)	1.02	0.87–1.21	0.78
RTC (×10^9^/L)	1.01	0.98–1.04	0.54
IRF (%)	1.07	0.97–1.18	0.16
EPO (mIU/mL)	1.10	1.04–1.16	<0.001 *
CRP (mg/L)	1.01	1.00–1.03	0.005 *
IL-6 (pg/mL)	1.02	1.01–1.03	0.001 *
ferritin/hepcidin	1.08	1.03–1.13	0.001 *
hepcidin/iron	1.07	1.00–1.15	0.037 *
hepcidin /CRP	0.80	0.61–1.06	0.12
hepcidin/IL-6	0.63	0.37–1.08	0.10
hepcidin/EPO	0.94	0.83–1.07	0.34

OR, odds ratio; CI, confidence interval; UIBC, unsaturated iron-binding capacity; TIBC, total iron-binding capacity; TSAT, transferrin saturation; sTfR, soluble transferrin receptors; RET-He, reticulocyte haemoglobin equivalent; RTC, reticulocytes; IRF, immature reticulocyte fraction; EPO, erythropoietin; CRP, C-reactive protein; IL-6, interleukin 6. * statistically significant results.

**Table 6:** Multiparameter model assessed by multivariable logistic regression analysis.

Odds for Critical COVID-19			
Parameter	OR	95% CI	*p*
EPO	1.10	1.04–1.16	<0.001
ferritin/hepcidin	1.08	1.02–1.14	0.004
The analysis gave results with 88% of correctly classified cases and AUC of 0.838 (0.749–0.905).			

OR, odds ratio; CI, confidence interval; EPO, erythropoietin. Data were considered significant if *p* < 0.05.

## Data Availability

Data are available from the corresponding author upon reasonable request.
